# Oropharyngeal candidiasis in HIV/AIDS patients and non-HIV subjects in the Southeast of Iran

**DOI:** 10.18502/cmm.4.4.379

**Published:** 2018-12

**Authors:** Abbas Hosain Pour, Samira Salari, Pooya Ghasemi Nejad Almani

**Affiliations:** 1 Neyriz Health Network, Shiraz University of Medical Sciences, Shiraz, Iran; 2 Student Research Committee, Kerman University of Medical Sciences, Kerman, Iran; 3 Department of Medical Parasitology and Mycology, Kerman University of Medical Sciences, Kerman, Iran; 4 Leishmaniasis Research Center, Kerman University of Medical Sciences, Kerman, Iran

**Keywords:** Candida species, HIV infection, Iran, Kerman, Oral cavity

## Abstract

**Background and Purpose:**

*Candida* species are the common opportunistic pathogens during the course of human immunodeficiency virus (HIV) infection. Oropharyngeal candidiasis (OPC) is generally known as the initial sign of HIV infection. The aim of this study was to compare demographic characteristics and frequency of *Candida* species between HIV/AIDS patients and non-HIV subjects in Kerman, southeast of Iran.

**Materials and Methods:**

This study was conducted on 143 samples collected from the oral cavity of 81 HIV/AIDS patients and 35 non-HIV subjects. The samples were cultured on Sabouraud dextrose agar and CHROMagar. The identification of *Candida* species was accomplished using the color of colony and polymerase chain reaction-restriction fragment length polymorphism.

**Results:**

According to the results, C. albicans (n=25, 69.14%) was the most prevalent species isolated from the HIV/AIDS patients, followed by C. glabrata (n=19, 23.46%). Other isolated species included *C. parapsilosis* (n=4, 4.94 %), *C. krusei *(n=1, 1.24%), and *C. kefyr* (n=1, 1.24%). Out of the 35 *Candida* species recovered from the oral samples of non-HIV subjects, 23 (65.71%) and 12 (34.29%) cases were *C. krusei *and *C. albicans*, respectively. *Candida* *krusei* was the only non-*albicans* species found in the non-HIV subjects that was also the predominant isolated species. Regarding the HIV/AIDS patients, the highest prevalence of OPC was observed in the age group of 41-50 years. However, in the non-HIV subjects, the age group of 31-40 years had the highest prevalence of this infection. Furthermore, no correlation was observed between the gender and number of *Candida* isolates.

**Conclusion:**

Consideration of the epidemiologic data showed that the two groups were significantly different in terms of the prevalence of *Candida* species, which could play a major role in the selection of effective drugs for the treatment of candidiasis.

## Introduction

Acquired immunodeficiency syndrome (**AIDS)** is an important dreadful disease in humans. Human immunodeficiency virus (HIV) is an agent, which may cause an infection. AIDS is the advanced condition of HIV infection that could take 10-15 years to develop. HIV is a chronic disease that can be managed, but not treated (1). AIDS weakens the immune system and makes the affected individuals susceptible to a number of conditions, such as renal and cardiovascular diseases, cancer, metabolic bone disease, lipodystrophy, vitamin D deficiency, a variety of opportunistic infections (e.g., viral, parasitic, fungal, and bacterial infections), and malignancies. These opportunistic diseases enhance the risk of mortality and particularly decrease the quality of life and life expectancy (2, 3). 

Candidiasis is a prevalent opportunistic infection in HIV/AIDS patients. This infection is caused by various *Candida* species, especially *C. albicans,* in the mouth, throat, and esophagus (4-6). Oropharyngeal candidiasis (OPC) is generally known as the initial manifestation of HIV infection. This condition usually develops in HIV-infected patients when the CD4^+^ T-lymphocyte count declines to > 350 CFU/ml. In the HIV patients with CD4 cell counts of ≤ 200 CFU/ml, thrush diffuses to the esophagus, thereby turning the oral candidiasis to esophageal candidiasis (7). 

Various types of OPC manifestations include pseudomembranous (thrush), erythematous, hyperplastic, and angular cheilitis, as well as mucocutaneous candidiasis (8, 9). The patterns of OPC in HIV-positive patients include pseudomembranous candidiasis, erythematous candidiasis, angular cheilitis, linear gingival erythema, ulcerations, oral hairy leukoplakia, and salivary gland swellings (10). 

The OPC is generally treated with antifungal agents depending on the severity of the infection. In this regard, the patients with mild to moderate OPC infections are usually managed with the oral administration of miconazole, clotrimazole, or nystatin for 1-2 weeks. Furthermore, the severe infection cases are generally prescribed fluconazole or another type of antifungal drug (11). With this background in mind, the present study was conducted to compare demographic characteristics, strains, and prevalence of *Candida* species between HIV-infected patients and non-HIV subjects in Kerman, southeast of Iran.

## Materials and Methods

***Subjects and sample collection***

This study was conducted on 81 HIV/AIDS patients and 35 non-HIV subjects from March 2017 to April 2018 (i.e., over 14 months) in Kerman. The HIV/AIDS cases were selected out of the patients referring to the Kerman Counseling Resource Center for Behavioral Disorders for receiving periodic examinations and antiviral drugs or solving various health problems. 

After making coordination with the Health Department of Kerman University of Medical Sciences, oral samples were collected from the patients. The investigated patients had not received prophylactic antifungal medications. They were checked for OPC signs and symptoms, including erythema with or without soreness or burning sensation, dysphagia, white patches, redness and soreness at the corners of the mouth. 

The non-HIV subjects were chosen out of the individuals referring to the Medical Mycology Laboratory of Afzalipoor Faculty of Medicine in Kerman. Samples were obtained from the oral cavity of the HIV-negative subjects that did not display any clinical signs or symptoms of OPC. Informed consent was obtained from all participants. They also filled out a structured questionnaire addressing demographic factors (e.g., age, gender, and personal health status) and duration HIV/AIDS. 

The samples were collected by scraping the subject’s oral mucosa and tongue with a sterile swab. All oral swabs were transported to the Mycology Research Laboratory without any delay and prepared for laboratory investigations on the same day. This study was approved by the Ethics Committee of Kerman University of Medical Sciences, Kerman (IR.KMU.REC.1395.146).

***Direct microscopic examination, staining, and culturing***

All the samples were examined in 10% potassium hydroxide (Merck, Germany). A portion of the samples were inoculated onto CHROMagar *Candida* medium (HiMedia, Mumbai, India) and Sabouraud dextrose agar (SDA, Merck, Germany), and then incubated at 37°C for at least 1 week.

***DNA extraction and molecular identification of different Candida species using polymerase chain reaction-restriction fragment length polymorphism***

In the present study, the identification of *Candida* species was accomplished through polymerase chain reaction-restriction fragment length polymorphism (PCR- RFLP) using a specific restriction enzyme, namely *Msp* I (Fermentas Life Sciences, Lithuania). Briefly, DNA was extracted from each of the *Candida* cultures obtained from HIV-infected and non-HIV subjects using Exgene Tissue SV Plus-mini kit (Gene All, General Bio System, South Korea) according to the manufacturer's instruction. 

The PCR amplification was performed using the universal fungal primers (i.e., ITS1: 5-TCC-GTAGGTGAA-CCT-GCG-G-3 and ITS4: 5-TCC-TCC-GCT-TAT-TGA-TATGC30; Pishgam Co, Tehran, Iran). Then, RFLP was implemented following the manufacturer's instructions using *Msp* I for the identification of different *Candida* species (12, 13). Finally, after the electrophoresis, *Candida* species were detected based on the size and number of the obtained bands (14).

***Statistical analysis***

The data were expressed as frequencies and percentages. Data analysis was performed in SPSS software, version 21 (SPSS, Inc. Chicago, Illinois) using Student’s paired t-test. 

## Results

***Demographic characteristic***

A total of 116 subjects, including 81 HIV/AIDS patients and 35 non-HIV subjects, were enrolled in this study. Table 1 presents the demographic characteristics of HIV-infected and non-HIV subjects associated with OPC. The mean ages of the HIV/AIDS and non-HIV subjects were 40.8±11.14 and 40.97±10.2 years, respectively. The results revealed no significant difference between the two groups in terms of age.

***Prevalence of Candida species in HIV-infected and non-HIV subjects***

**Figure 1:**
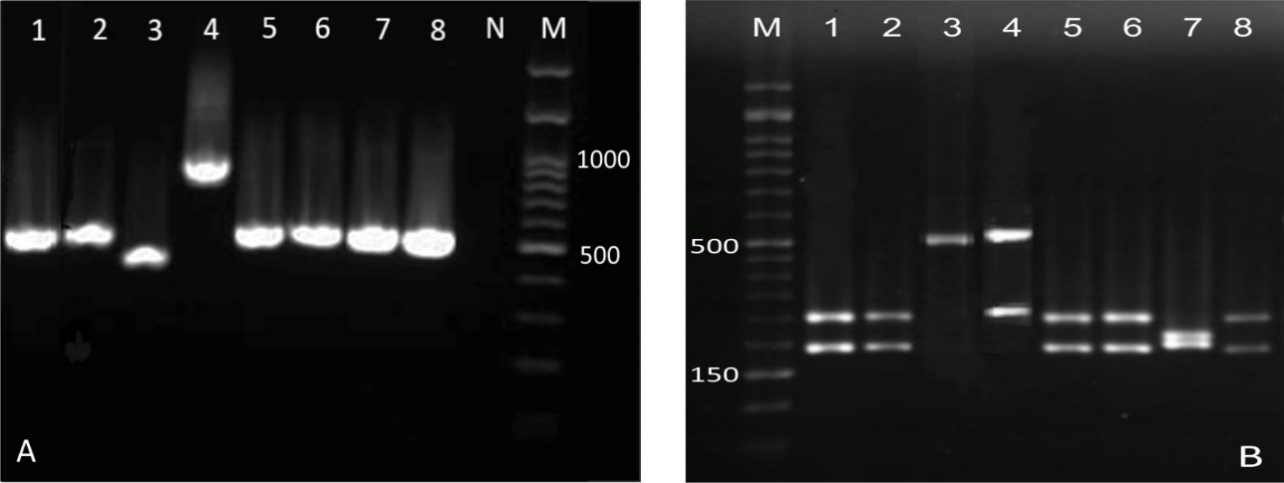
Patterns of polymerase chain reaction products of *Candida *isolates before (A) and after (B) digestion with *Msp I* in HIV/AIDS patients; in figure B: lanes 1, 2, 5, 6, and 8: *C. albicans *(297 and 238 bp), lane 3: *C. parapsilosis *(520 bp), lane 4: *C. glabrata *(557 and 341 bp), lane 7: *C. krusei *(261 and 249 bp), and lane M: 50 bp ladder

Figure 1 displays the patterns of ITS-RFLP for *Candida *species before and after digestion with *Msp I *in HIV/AIDS patients. A total of 81 *Candida* isolates were obtained from the oral samples of HIV/AIDS patients. Figure 1 also depicts the prevalence of different *Candida* species among HIV-infected and non-HIV subjects. The analysis led to the identification of five different species, the most common of which was *C. albicans*. 

**Figure 2:**
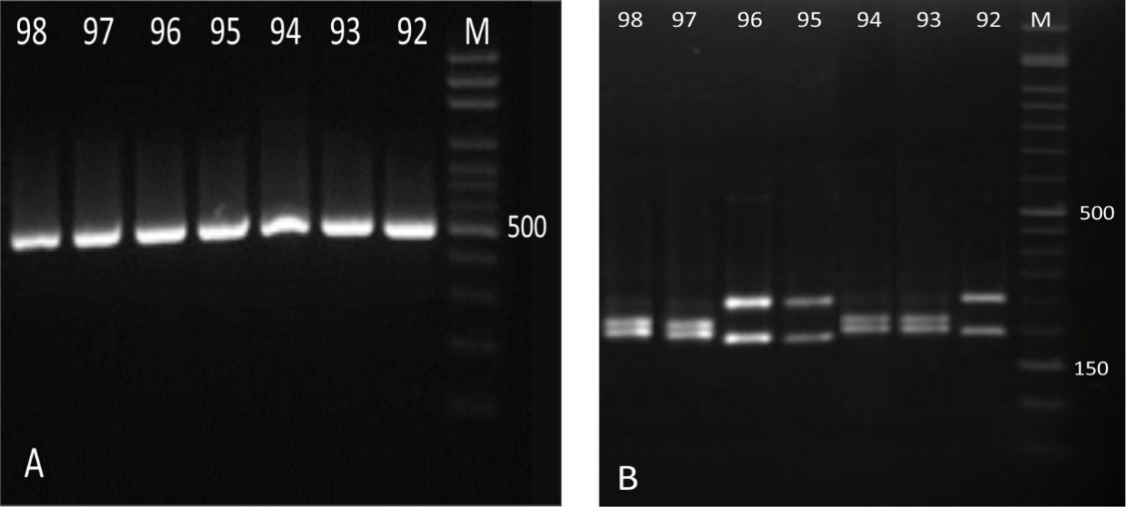
Pattern of polymerase chain reaction products of *Candida* isolates before (A) and after (B) digestion with *Msp I* in non-HIV subjects; in figure B: lanes 92, 95, and 96: *C. albicans *(297 and 238 bp), lanes 93, 94, 97, and 98: *C. krusei *(261 and 249 bp), lane M: 50 bp ladder

**Figure 3:**
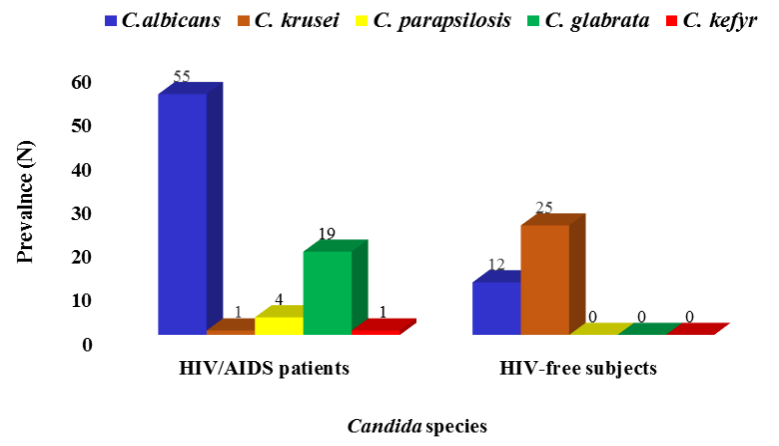
Prevalence of *Candida* species in HIV/AIDS patients and non-HIV subjects

Figure 2 illustrates the patterns of ITS-RFLP for *Candida* species before and after digestion with *Msp I* in non-HIV subjects. *Candida* *krusei* was the only non-*albicans* species found in non-HIV subjects and identified as the predominant isolated species. Nevertheless, no *C. glabrata*, *C. parapsilosis*, or *C. kefyr* were detected in these subjects (Figure 2). There was a statistically significant difference between the HIV/AIDS patients and non-HIV subjects in terms of prevalence of identified *Candida* species (Figure 3).

**Table 1:** Demographic characteristics of HIV/AIDS patients and non-HIV subjects

**Characteristics**	**Classification**	**HIV/AIDS patients (n=81)**		**non-HIV subjects (n=35)**	
		**Number (%)**	**95% CI**	**Number (%)**	**95% CI**
Age groups	0-10	1 (1.235)	0.06177-6.089	0 (0.0)	-
	11-20	4 (4.938 )	1.569-11.91	0 (0.0)	-
	21-30	5 (6.173)	2.262-13.68	6 (17.14)	8.1-32.68
	31-40	20 (24.69)	16.59-35.08	13 (37.14)	23.16-53.66
	41-50	38 (46.91)	36.43-57.67	10 (28.57)	16.33-45.05
	51-60	11 (13.58)	7.76-22.7	6 (17.14)	8.1-32.68
	61-70	2 (2.47)	0.68-8.56	0 (0.0)	-
Gender	Male	29 (35.8)	53.34-73.78	27 (77.14)	60.98-87.93
	Female	52 (64.2)	26.22-46.66	8 (22.86)	12.07-39.02
Duration of HIV/AIDS (years)	0-1	6 (7.41)	3.44-15.24	0 (0.0)	
	1-5	32 (39.51)	29.57-50.4		
	5-10	22 (27.16)	18.67-37.71		
	10-15	15 (18.52)	11.56-28.33		
	≥ 15	6 (7.41)	3.44-15.24		

Table 2 shows the frequency of the *Candida* species recovered from oral candidiasis in HIV/AIDS patients. Based on the results, the frequency of the *Candida* species was significantly higher in females compared with that in males (64.2% vs. 35.8%). However, in non-HIV subjects with OPC, the prevalence of *Candida* species was four times higher in men than in women (80% vs. 20%). No correlation was observed between gender and number of *Candida* species (Table 2).

**Table 2:** Distribution of *Candida* species in HIV/AIDS patients and non-HIV subjects with oropharyngeal candidiasis based on gender

***Candida*** ** species**	**Male n (%)**		**Female n (%)**		**Total n (%)**		**95% CI**		***P-value***
	**HIV/AIDS patients**	**non-HIV subjects**	**HIV/AIDS patients**	**non-HIV subjects**	**HIV/AIDS patients**	**non-HIV subjects**	**HIV/AIDS patients**	**non-HIV** ** subjects**	
*C. albicans*	21 (25.93)	10 (28.57)	35 (43.21)	2 (5.7)	56 (69.14)	12 (34.29)	58.41-7814	20.84-50.85	0.11
*C. krusei*	0 (0.0)	18 (51.43)	1 (1.235)	5 (14.29)	1 (1.235)	23 (65.71)	0.062-6.089	49.15-79.16	0.23
*C. parapsilosis*	2 (2.47)	0 (0.0)	2 (2.47)	0 (0.0)	4 (4.938 )	0 (0.0)	1.569-11.91	-	0.18
*C. glabrata*	5 (6.173)	0 (0.0)	14 (17.28)	0 (0.0)	19 (23.46)	0 (0.0)	15.57-33.76	-	0.16
*C. kefyr*	1 (1.235)	0 (0.0)	0 (0.0)	0 (0.0)	1 (1.235)	0 (0.0)	0.062-6.089	-	0.42
Total	29 (35.8)	28 (80)	52 (64.2)	7 (20)	81 (100)	35 (100)	100	100	-

## Discussion

The aim of this study was to compare demographic characteristics, strains, and prevalence of *Candida* species in HIV-infected and non-HIV subjects in Kerman. In Kerman province, 729 cases of AIDS have been identified, 90% of whom are male. About 75% of these patients had been infected via injecting drug, while 19% of them had a history of high-risk sexual activity, and 2% of them had been infected through the mother-to-child transmission. 

The prevalence of HIV/AIDS among women has undergone an increase in recent years. In the present study, OPC was more prevalent in females than in males. As our results indicated, the age group of 41-50 years had the maximum *Candida* species frequency (n=38, 46.91%), rendering a male to female ratio of 1.79:1. Maheshwari et al. (3) reported that the age group of 21-45 years had the highest prevalence of OPC with a male to female ratio of 2.2:1. They suggested that some factors, such as occupation and higher rate of migration, predispose the males to such infection, compared to females. 

The OPC has been reported to be prevalent among the HIV/AIDS patients in the age group of 29-39 years (15). Similar to our results, Lar *et al.* (16) and Awoyeni *et al*. (15) reported a higher rate of OPC in women than in men. However, unlike our findings, in another study, HIV patients with the age group of 61-70 years had the highest prevalence of OPC. It seems that the high prevalence of OPC in the elderly could be due to their weaker immune system (17). 

The difference in OPC prevalence between genders could be related to their lack of awareness about the risk factors for virus transmission from sexual partner, drug injection, or non-use of condom. The weakening of the immune system by the virus sets the condition for the growth of *Candida* species, especially OPC development. The males reportedly show less tendency to refer to the special health centers for undergoing HIV test, counseling, and routine examinations until the disease becomes symptomatic (15).

Oral carriage of Candida species (62-67%) is prevalent in HIV/AIDS patients (2). Our results showed that 69.14% of the oral samples obtained from these patients were *C. albicans *and 30.86% of the cases belonged to non-*Candida* *albicans* *Candida* (NCAC) species. These findings are in agreement with the those obtained by Katiraee *et al.* (18), Li *et al. *(19), and Mousavi *et al.* (20). Nonetheless, Kwamin^,^s *et al.* (21) and Maheshwari *et al.* (3) reported lower isolation rates of* C. albicans* in HIV/AIDS patients than our study. 

In line with our research, in a study performed in India, 61.7% of the *Candida* isolates obtained from HIV/AIDS patients were C*. albicans*. However, in the mentioned study, the frequency and diversity of NCAC species were different from our results. In contrast to the present study, the common NCAC species were *C*. guilliermondii (n=14) and C. parapsilosis (n=9) (4). Nonetheless, no cases of *C.* *guilliermondii*, C. tropicalis, C. dubliniensis, or C. famata *were found in the current study. *

The frequency of *Candida* carriage among the non-HIV subjects was obtained as 40%. Four different *Candida* species were identified in this group, the most common of which were *C. albicans, *followed by C. tropicalis (22). Consistent with our results, in Yitayew^,^s study, HIV/AIDS patients were reported to have a significantly higher frequency of oral *Candida* species carriage as compared to non-HIV subjects (23). Here, the most common *Candida* species recovered from non-HIV subjects was* C. krusei,* followed by *C. albicans*. 

The discrepancy in the reported frequency and diversity of identified *Candida* species could be related to the difference in sampling methods, sample size, demographic and clinical characteristics of studied population, such as difference in T CD_4_ count, high-risk behaviors (e.g., high-risk sexual behaviors, improper diet, and smoking), oral hygiene, lifestyle, intravenous drug abuse, use of highly active antiretroviral therapy, having dentures, antifungal therapy, immune system, and geographical locations (19, 23, 24). 

One of the limitations of the present study was the non-cooperation of the affected people for sampling or filling the questionnaire. Furthermore, the size of the two studied groups was unequal. Another limitation was the incapability of PCR-RFLP to discriminate some species, such as *C. parapsilosis* complex and more importantly *C. dubliniensis*, which is a common identified species in patients with HIV/AIDS.

## Conclusion

The OPC is known as a major opportunistic infection among HIV/AIDS patients. This study compared the demographic characteristics, strains, and prevalence of *Candida* species in HIV-infected and non-HIV subjects in Kerman. Antiretroviral and antifungal therapies could change the frequency and distribution of *Candida* species.
